# Supplementary material for: More than 17,000 tree species are at risk from rapid global change
Source: Nat Commun. 2024 Jan 2;15:166. doi: 10.1038/s41467-023-44321-9 (PMC10761716; doi:10.1038/s41467-023-44321-9)
Supplement: Supplementary file 1 — Supplementary Information [file 41467_2023_44321_MOESM1_ESM.pdf]

# Supplementary Information

## More than 17,000 tree species are at risk from rapid global change

Authors:

Coline C.F. Boonman, Josep M. Serra-Diaz, Selwyn Hoeks, Wen-Yong Guo, Brian J. Enquist, Brian Maitner, Yadvinder Malhi, Cory Merow, Robert Buitenwerf, Jens-Christian Svenning

**Table 1. Overview of highly exposed tree species per conservation status group** Values indicated in column 3 depict the lower and upper threshold of harmful rates of recent change (RRC; year$^{-1}$) defined as the 5$^{th}$ and 95$^{th}$ quantile. Note that the total number of prioritized species (*n*) includes species that occupied minimal areas (*n*=9,741), had a line as minimum convex polygon (n = 3), or had an outlier RRC value (*n* = 1).

| Threats | | Threshold of RRC | All species | Threatened | Vulnerable | Not Threatened | Data Deficient | Not Evaluated |
|---|---|---|---|---|---|---|---|---|
| | | [year$^{-1}$] | (*n*=32,090) | (*n*=2,418) | (*n*=2,710) | (*n*=16,503) | (*n*=540) | (*n*=9,919) |
| | | 5$^{th}$ quantile 95$^{th}$ quantile | | | | | | |
| Cropland expansion | | > 0.50% of extent | 1605 | 133 | 173 | 717 | 16 | 566 |
| Tree cover decline | | > 3.55% of extent | 1605 | 286 | 240 | 455 | 37 | 587 |
| Built-up expansion | | > 0.23% of extent | 1605 | 89 | 93 | 855 | 32 | 536 |
| Deforestation | | > 1.85% of extent | 1605 | 239 | 275 | 599 | 26 | 466 |
| Burned area | | < -0.24% of extent | 1605 | 82 | 137 | 1045 | 30 | 311 |
| | | > 0.04% of extent | 1605 | 166 | 207 | 777 | 23 | 432 |
| Climate Change, Comprising: | Minimum Temperature | < 0 °C | 965 | 42 | 56 | 389 | 16 | 462 |
| | | > 0.06 °C | 1605 | 109 | 77 | 859 | 45 | 515 |
| | Maximum Temperature | < 0 °C | 1419 | 107 | 118 | 774 | 15 | 405 |
| | | > 0.09 °C | 1605 | 146 | 141 | 688 | 70 | 560 |
| | VPD | < 0 Pa | 852 | 128 | 99 | 419 | 10 | 196 |
| | | > 7.14 Pa | 1605 | 134 | 141 | 733 | 58 | 539 |
| | VPD seasonality | < -0.67 Pa | 1605 | 170 | 186 | 647 | 27 | 575 |
| | | > 3.33 Pa | 1605 | 89 | 101 | 728 | 49 | 638 |
| | Precipitation | < -3.77 mm | 1605 | 204 | 205 | 625 | 23 | 548 |
| | | > 2.05 mm | 1605 | 201 | 193 | 453 | 29 | 729 |
| | Precipitation seasonality | < -2.20 mm | 1605 | 206 | 205 | 502 | 27 | 665 |
| | | > 1.78 mm | 1605 | 225 | 215 | 512 | 15 | 638 |

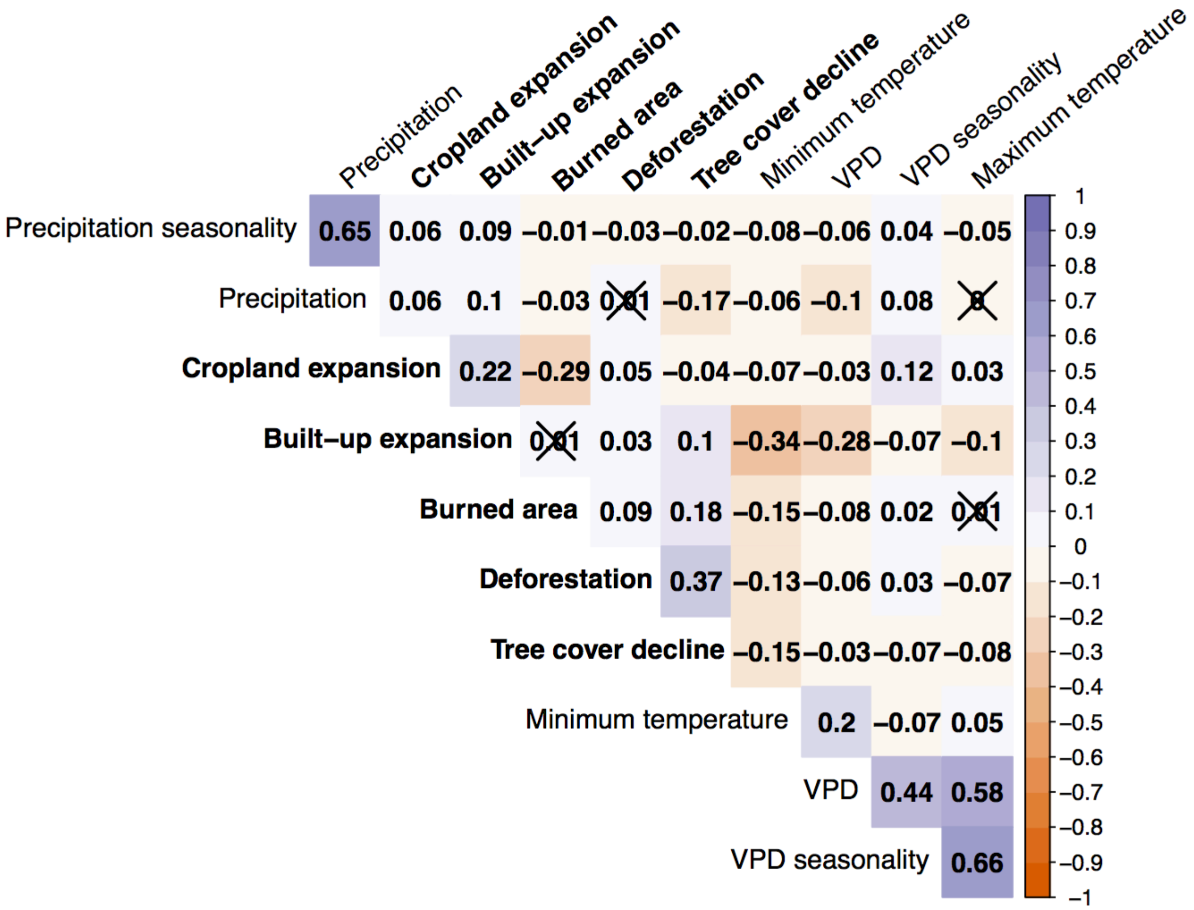


**Figure 1. Correlation matrix between the rates of recent change values** of the different threats for all tree species. All main threats are presented in bold, whereas components of the sixth threat, Climate Change, are not. Purple shades indicate positive correlations while orange shades indicate negative correlations. The numbers represent the r value of the correlation where insignificant correlations are indicated by a cross.

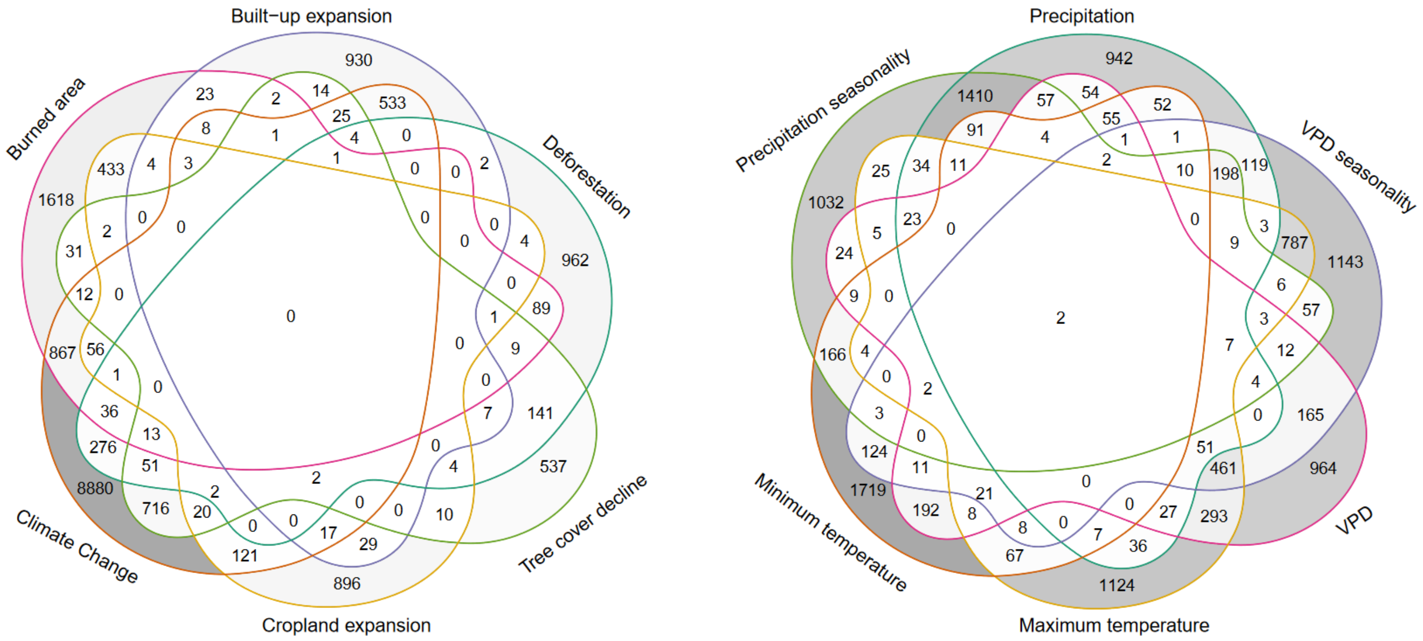


**Figure 2. Overlap of species between prioritization lists of different threats.** The left image represents the six main threats, whereas the right image presents the six components that comprise the Climate Change threat. Each threat, identified with a different color, had a list of priority candidates for IUCN Red List re-evaluation. Whenever a species occurred in more than one list, indicating multiple pressures for those species, it is indicated here. Numbers inside cells indicate the number of species, where darker shades indicate higher numbers.

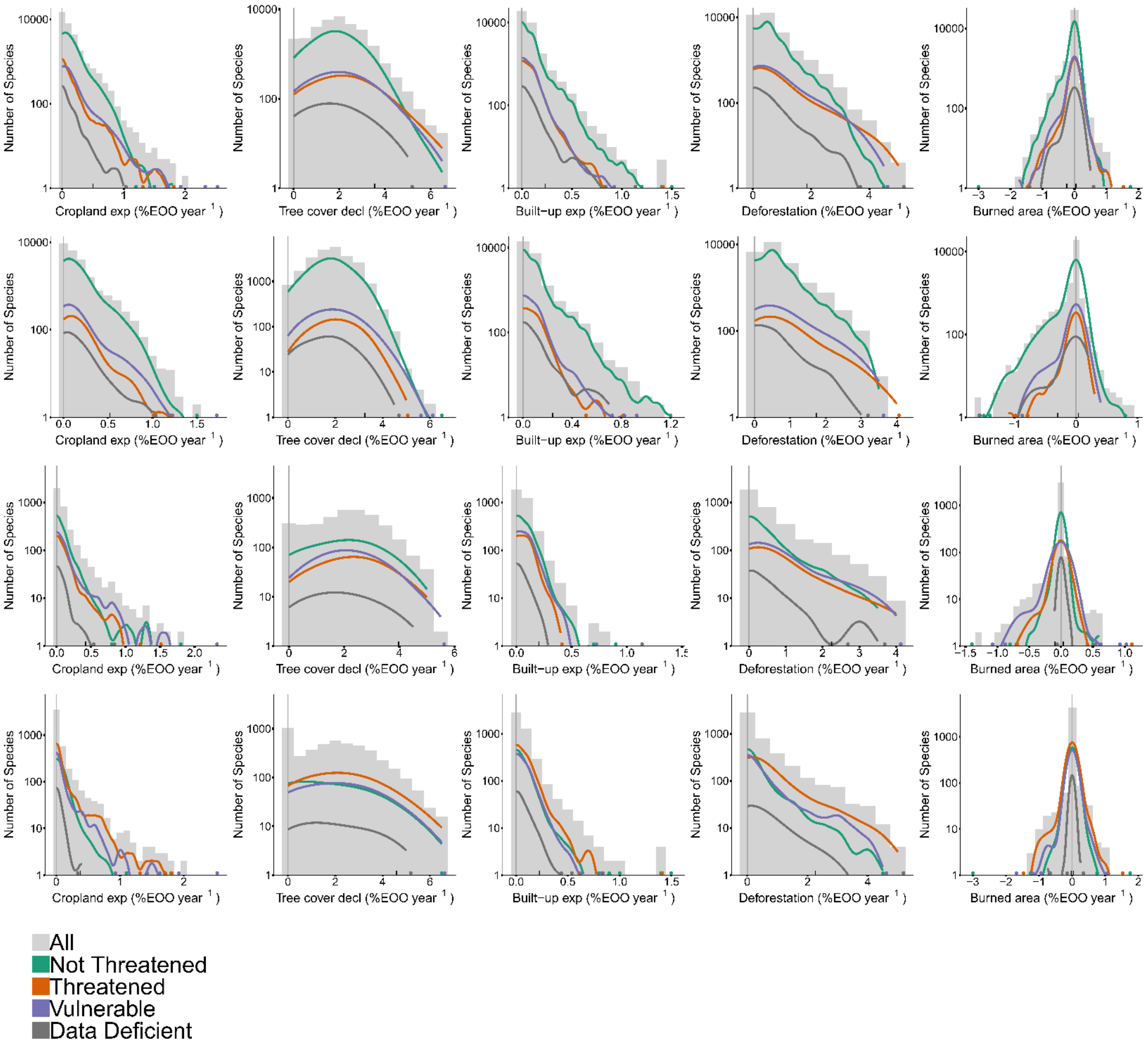


**Figure 3. Rates of recent change for five proxies of identified threats to trees** split up by the size of species' extent. The top row includes all species, and the second, third and fourth row include only species with a large-sized, medium-sized and small-sized extent, respectively (for size categorization, see Methods). The vertical grey line indicates no change (0 % of the extent year$^{-1}$). At the bottom of each plot, colored dots indicate rates of recent change per conservation status group when only one species has that value and black ticks identify the 5$^{th}$ and 95$^{th}$ quantile. Figures on the sixth threat, Climate Change, are below, separated per considered component.

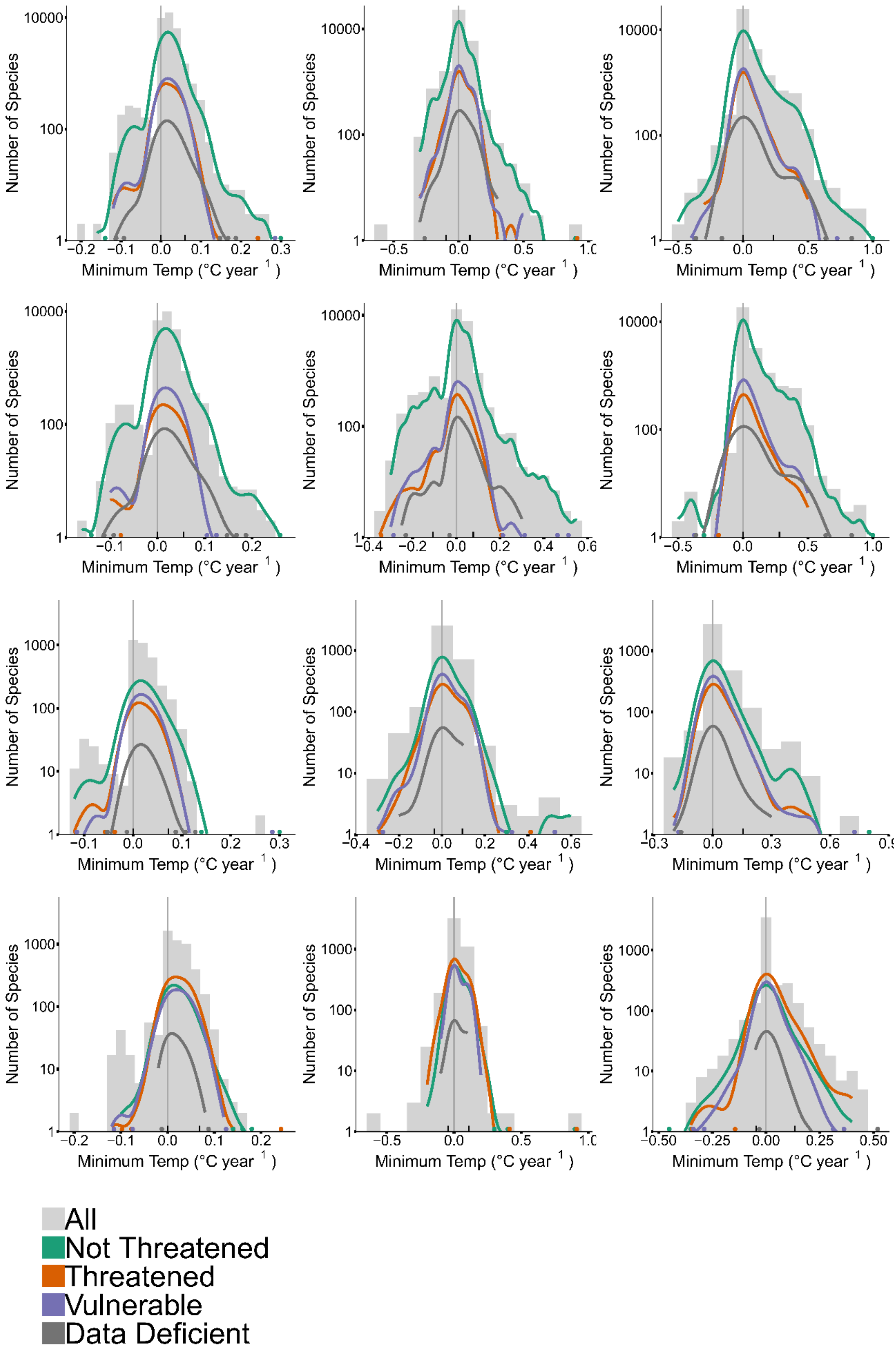


**Figure 4. Distribution of rates of recent change of minimum temperature (Minimum Temp)** split up by the size of species' extent and different time windows. The top row includes all species, and the second, third and fourth row include only species with a large-sized, medium-sized and small-sized extent, respectively (for size categorization, see Methods). The left column includes the 20-year time window, the middle column indicates changes for the 10-year time window from 2000-2010, and the right column indicates changes for the 10-year time window from 2010-2020. The vertical grey line indicates no change (0 °C year$^{-1}$). At the bottom of each plot, colored dots indicate rates of recent change per conservation status group when only one species has that value and black ticks identify the 5th and 95th quantile.

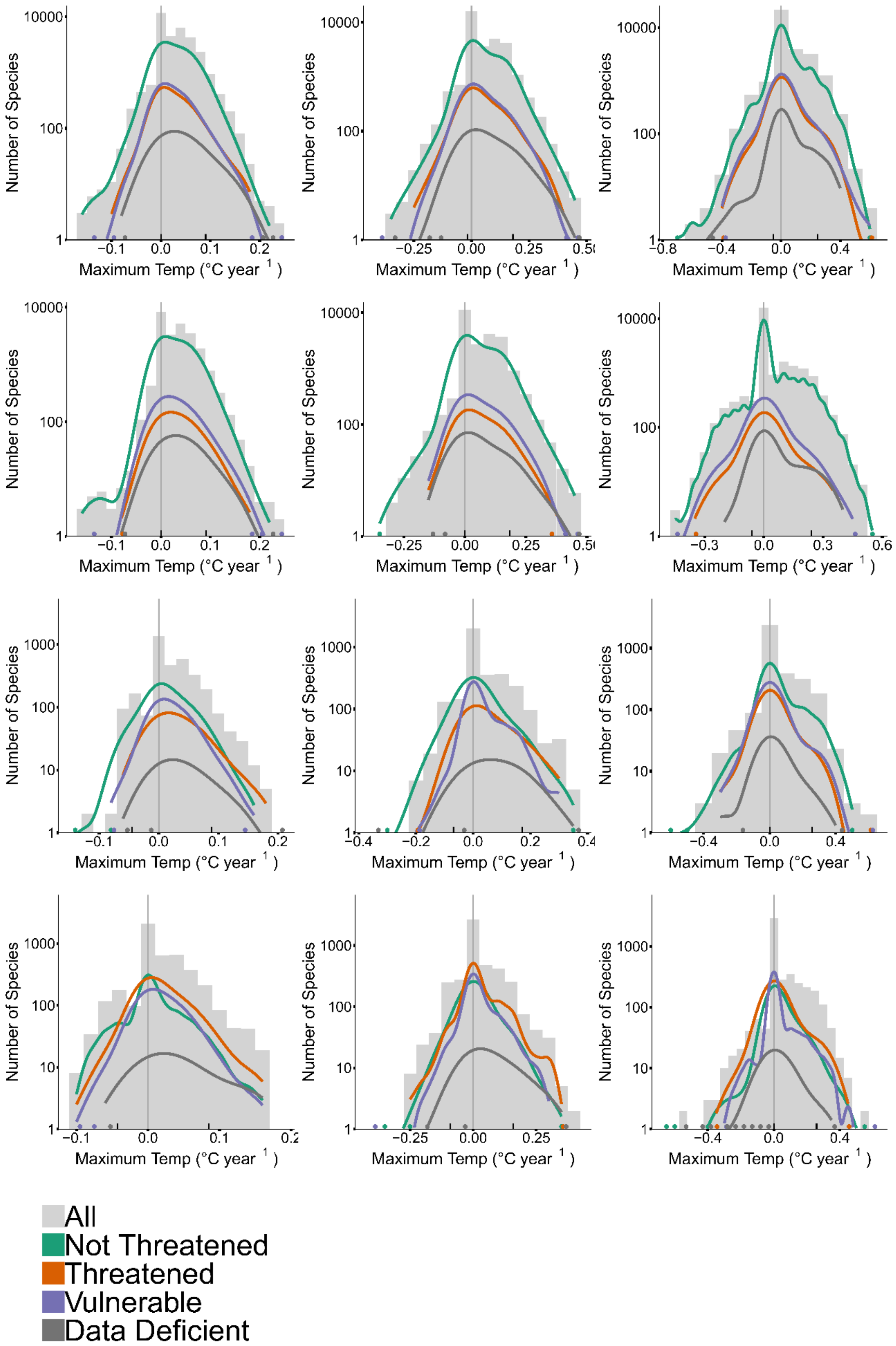


**Figure 5. Distribution of rates of recent change of maximum temperature (Maximum Temp)** split up by the size of species' extent and different time windows. The top row includes all species, and the second, third and fourth row include only species with a large-sized, medium-sized and small-sized extent, respectively (for size categorization, see Methods). The left column includes the 20-year time window, the middle column indicates changes for the 10-year time window from 2000-2010, and the right column indicates changes for the 10-year time window from 2010-2020. The vertical grey line indicates no change (0 °C year$^{-1}$). At the bottom of each plot, colored dots indicate rates of recent change per conservation status group when only one species has that value and black ticks identify the 5th and 95th quantile.

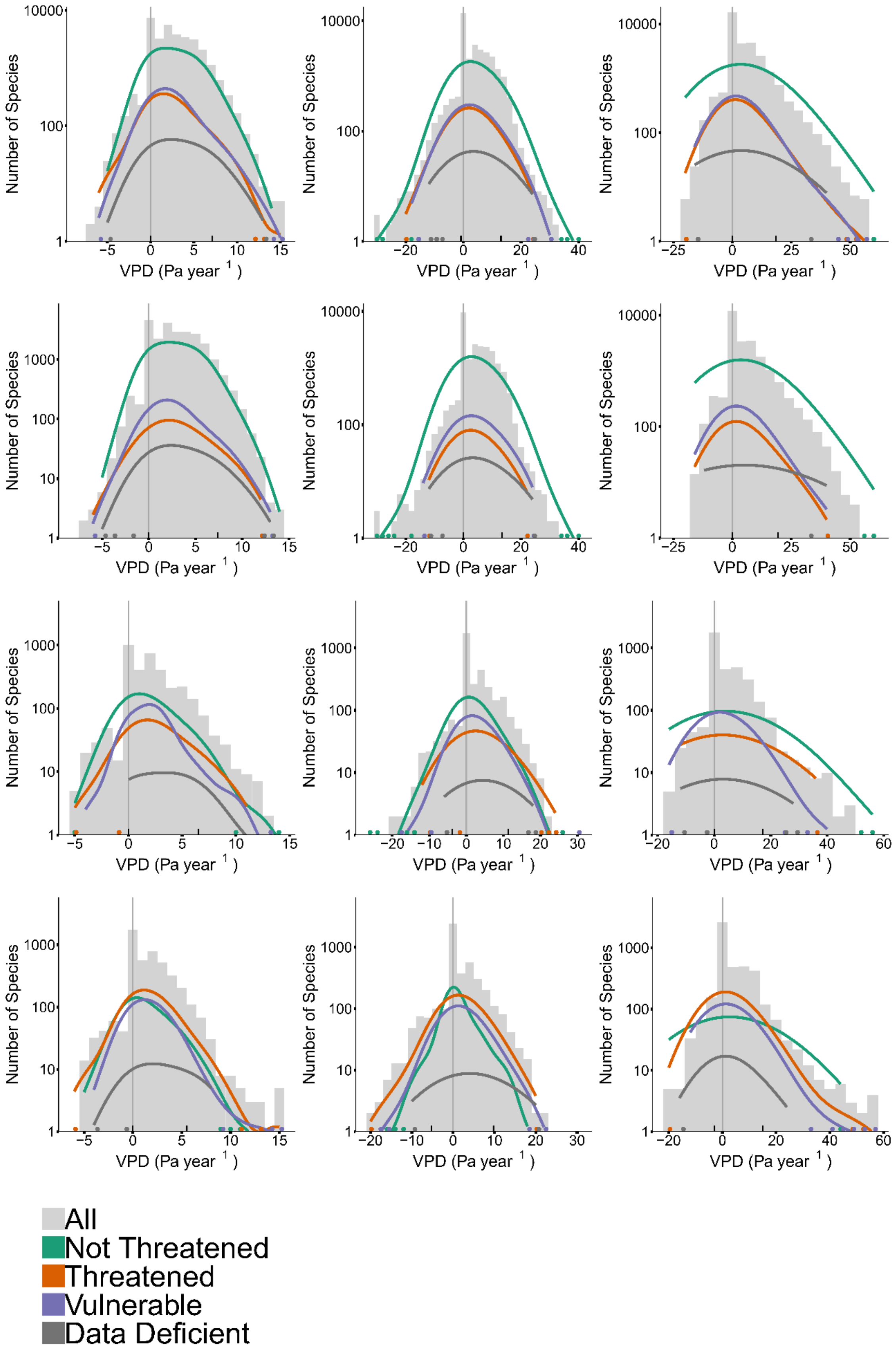


**Figure 6. Distribution of rates of recent change of VPD** split up by the size of species' extent and different time windows. The top row includes all species, and the second, third and fourth row include only species with a large-sized, medium-sized and small-sized extent, respectively (for size categorization, see Methods). The left column includes the 20-year time window, the middle column indicates changes for the 10-year time window from 2000-2010, and the right column indicates changes for the 10-year time window from 2010-2020. The vertical grey line indicates no change (0 Pa year$^{-1}$). At the bottom of each plot, colored dots indicate rates of recent change per conservation status group when only one species has that value and black ticks identify the 5th and 95th quantile.

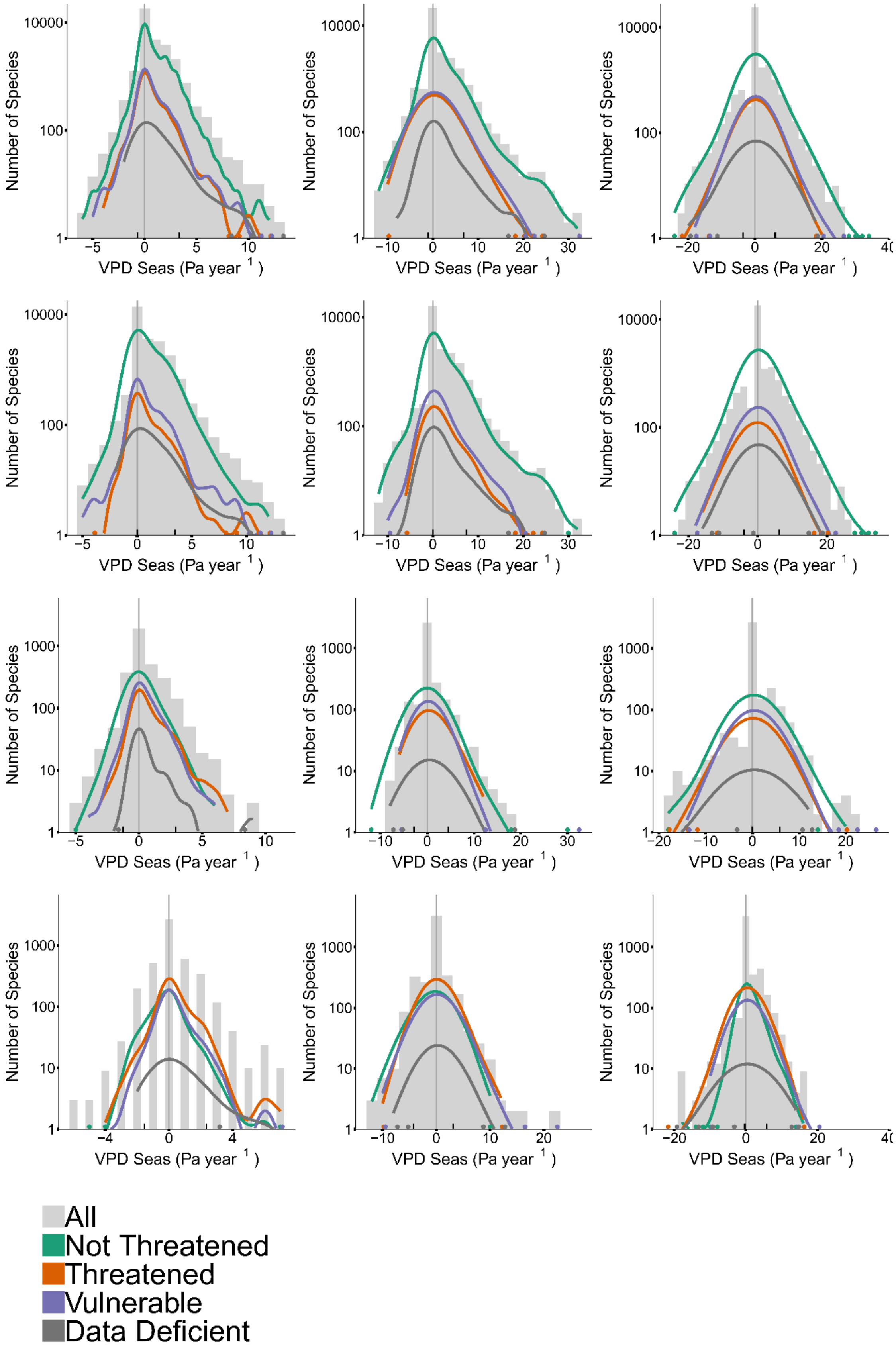


**Figure 7. Distribution of rates of recent change of VPD seasonality (VPD Seas)** split up by the size of species' extent and different time windows. The top row includes all species, and the second, third and fourth row include only species with a large-sized, medium-sized and small-sized extent, respectively (for size categorization, see Methods). The left column includes the 20-year time window, the middle column indicates changes for the 10-year time window from 2000-2010, and the right column indicates changes for the 10-year time window from 2010-2020. The vertical grey line indicates no change (0 Pa year$^{-1}$). At the bottom of each plot, colored dots indicate rates of recent change per conservation status group when only one species has that value and black ticks identify the 5th and 95th quantile.

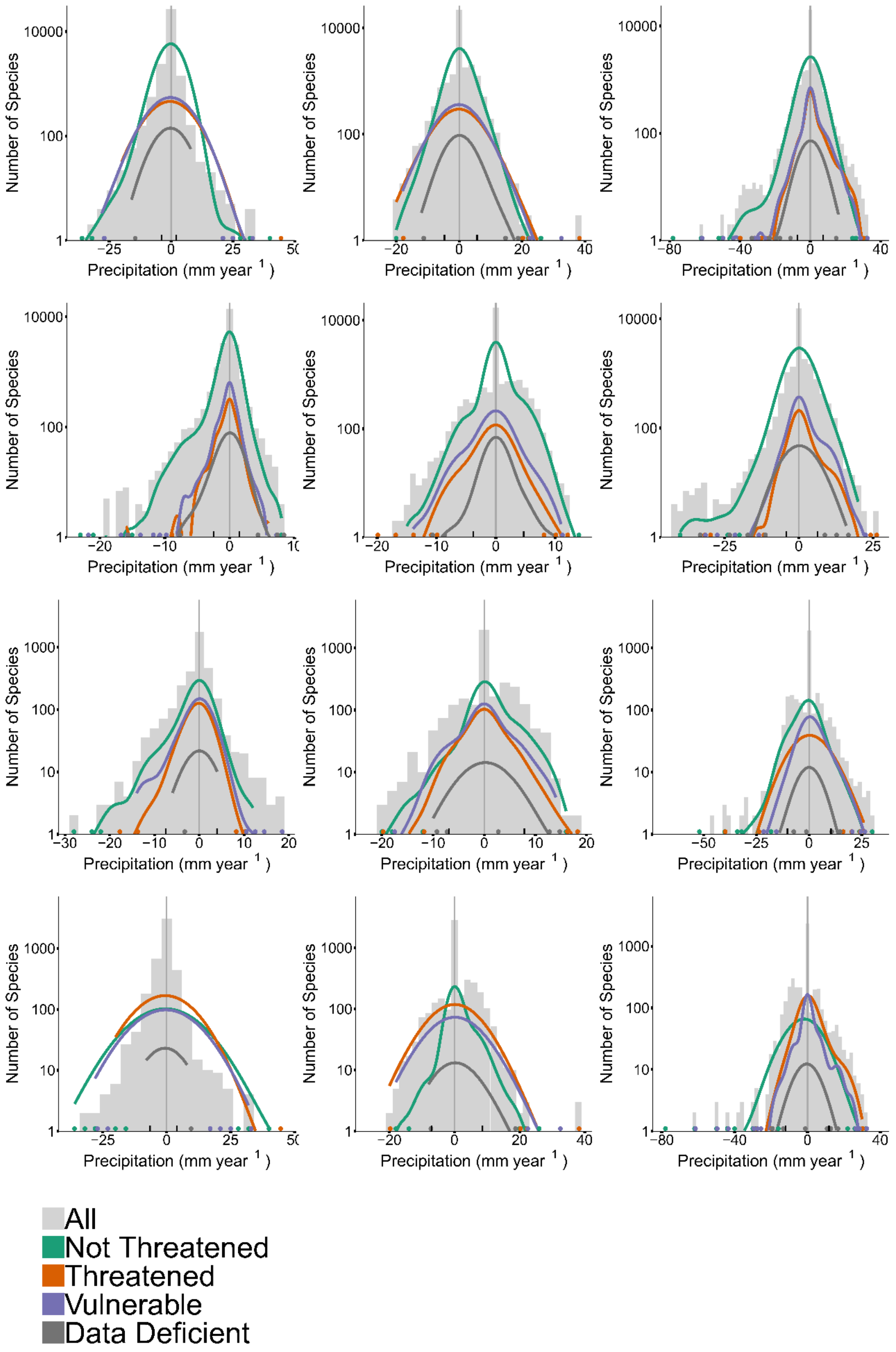


**Figure 8. Distribution of rates of recent change of precipitation** split up by the size of species' extent and different time windows. The top row includes all species, and the second, third and fourth row include only species with a large-sized, medium-sized and small-sized extent, respectively (for size categorization, see Methods). The left column includes the 20-year time window, the middle column indicates changes for the 10-year time window from 2000-2010, and the right column indicates changes for the 10-year time window from 2010-2020. The vertical grey line indicates no change (0 mm year$^{-1}$). At the bottom of each plot, colored dots indicate rates of recent change per conservation status group when only one species has that value and black ticks identify the 5th and 95th quantile.

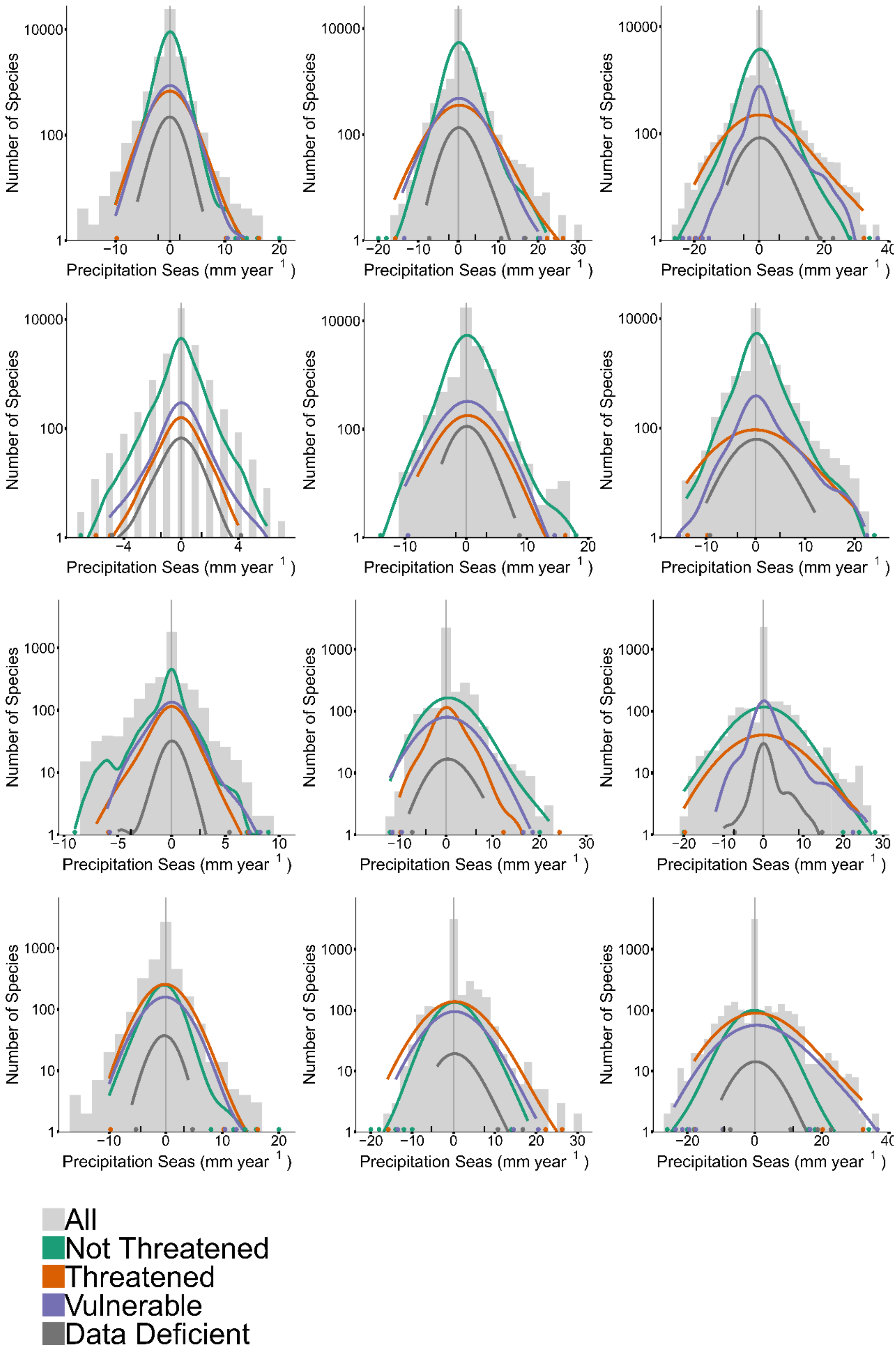


**Figure 9. Distribution of rates of recent change of precipitation seasonality (Precipitation Seas)** split up by size of species' extent and different time windows. The top row includes all species, and the second, third and fourth row include only species with a large-sized, medium-sized and small-sized extent, respectively (for size categorization, see Methods). The left column includes the 20-year time window, the middle column indicates changes for the 10-year time window from 2000-2010, and the right column indicates changes for the 10-year time window from 2010-2020. The vertical grey line indicates no change (0 mm year$^{-1}$). At the bottom of each plot, colored dots indicate rates of recent change per conservation status group when only one species has that value and black ticks identify the 5th and 95th quantile.

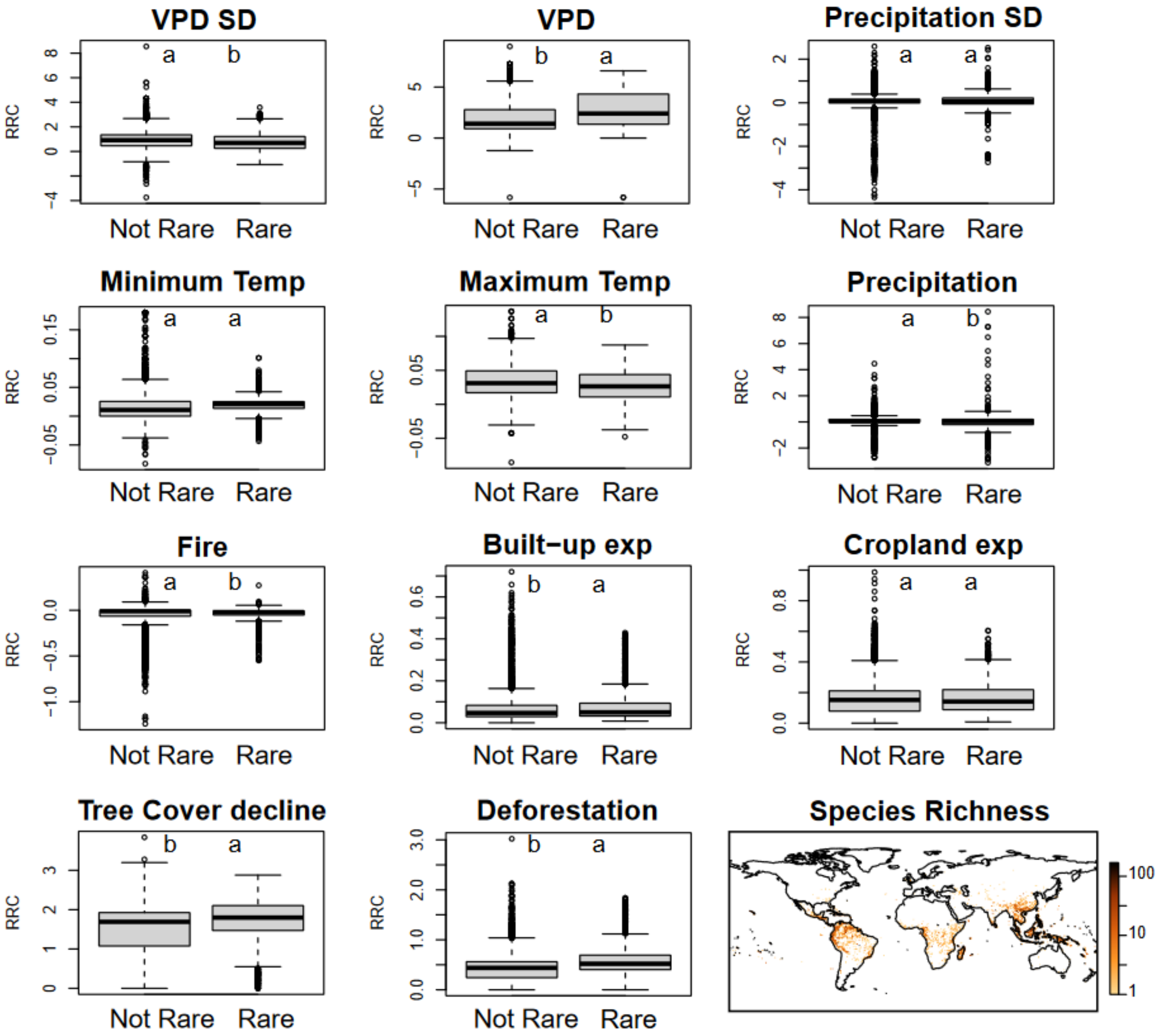


**Figure 10. Distribution of rates of recent change values (RRC) for rare and not rare species.** Here, rare species refer to grid cells where species occur that are excluded for the rates of recent change analysis because of their rarity (AOO < 10 $km^2$ or < 5 occurrence records, 9,741 species). In this figure, the locations of these species are labeled as 'Rare' and we compare the average rates of recent change values of the grid cells where 'Rare' species occur with average rates of recent change values of grid cells that do not include 'Rare' species, labeled as 'Not Rare'. Values are calculated as the average rate of recent change value per threat for all species occurring in those grid cells. The lower right image shows the number of excluded species per grid cell. Differences between groups of grid cells in terms of mean rate of recent change value according to the Welch two sample t-test with a significance level of $p < 0.001$ are indicated with letters, where a identifies the mean value with the larger difference from 0 and b identifies the mean value with the smaller difference from 0. The lower five plots are the main threat, while the top six plots are the components that comprise the climate change threat. Boxplots indicate the median, $1^{st}$ and $3^{rd}$ quantiles, and the whiskers present the minimum and maximum excluding outliers.

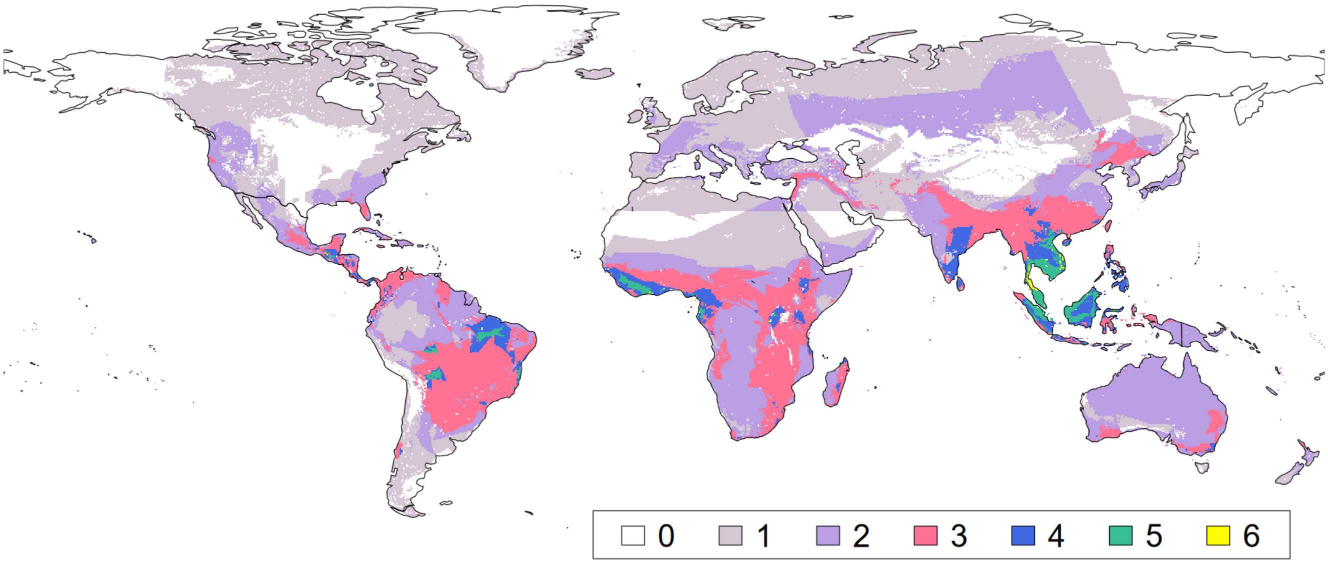


**Figure 11. Hotspots of multiple pressures.** After binarizing the maps of Figure 3 of the Main text, they were superimposed, identifying areas where species are exposed to high changes in multiple threats. The numbers identify the number of threats.

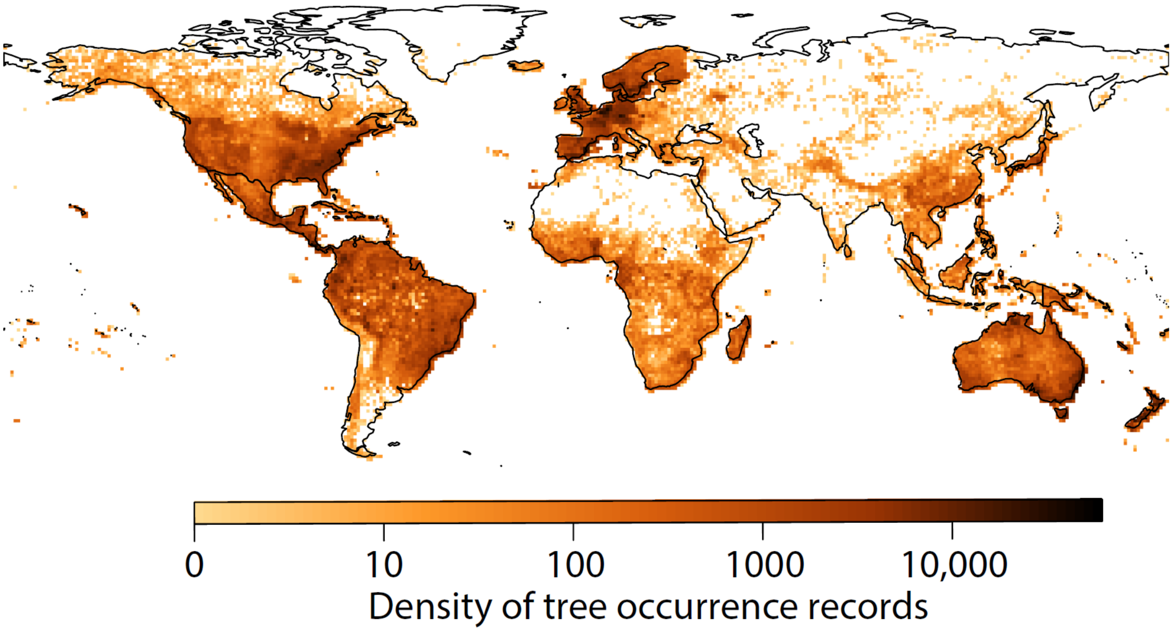


**Figure 12. Overview of all tree species occurrence records used in this study.** Colors indicate the density of observations in number of trees per 100 $km^2$ grid cells.
